# Malignant Tumor Purity Reveals the Driven and Prognostic Role of *CD3E* in Low-Grade Glioma Microenvironment

**DOI:** 10.3389/fonc.2021.676124

**Published:** 2021-09-07

**Authors:** Xiuqin Lu, Chuanyu Li, Wenhao Xu, Yuanyuan Wu, Jian Wang, Shuxian Chen, Hailiang Zhang, Huadong Huang, Haineng Huang, Wangrui Liu

**Affiliations:** ^1^Department of Nursing and Health Management, Shanghai University of Medicine & Health Sciences, Shanghai, China; ^2^Department of Neurosurgery, Affiliated Hospital of Youjiang Medical University for Nationalities, Guangxi, China; ^3^Department of Urology, Fudan University Shanghai Cancer Center, Shanghai Medical University, Fudan University, Shanghai, China; ^4^Department of Gastroenterology, Naval Medical Center of People’s Liberation Army (PLA) of China, Naval Military Medical University, Shanghai, China; ^5^Department of Transplantation, Xinhua Hospital Affiliated to Shanghai Jiao Tong University School of Medicine, Shanghai, China; ^6^Department of Oncology, Xinhua Hospital Affiliated to Shanghai Jiao Tong University School of Medicine, Shanghai, China

**Keywords:** tumor microenvironment, tumor purity, *CD3E*, low-grade glioma, prognosis, immune infiltrations

## Abstract

The tumor microenvironment (TME) contributes to the initiation and progression of many neoplasms. However, the impact of low-grade glioma (LGG) purity on carcinogenesis remains to be elucidated. We selected 509 LGG patients with available genomic and clinical information from the TCGA database. The percentage of tumor infiltrating immune cells and the tumor purity of LGG were evaluated using the ESTIMATE and CIBERSORT algorithms. Stromal-related genes were screened through Cox regression, and protein-protein interaction analyses and survival-related genes were selected in 487 LGG patients from GEO database. Hub genes involved in LGG purity were then identified and functionally annotated using bioinformatics analyses. Prognostic implications were validated in 100 patients from an Asian real-world cohort. Elevated tumor purity burden, immune scores, and stromal scores were significantly associated with poor outcomes and increased grade in LGG patients from the TCGA cohort. In addition, *CD3E* was selected with the most significant prognostic value (Hazard Ratio=1.552, *P*<0.001). Differentially expressed genes screened according to *CD3E* expression were mainly involved in stromal related activities. Additionally, significantly increased *CD3E* expression was found in 100 LGG samples from the validation cohort compared with adjacent normal brain tissues. High *CD3E* expression could serve as an independent prognostic indicator for survival of LGG patients and promotes malignant cellular biological behaviors of LGG. In conclusion, tumor purity has a considerable impact on the clinical, genomic, and biological status of LGG. *CD3E*, the gene for novel membrane immune biomarker deeply affecting tumor purity, may help to evaluate the prognosis and develop individual immunotherapy strategies for LGG patients. Evaluating the ratio of differential tumor purity and *CD3E* expression levels may provide novel insights into the complex structure of the LGG microenvironment and targeted drug development.

## Background

The treatment and prognosis of glioma are relatively limited because the understanding of immune gene regulation and carcinogenesis is incomplete (1, 2). In the United States, the annual incidence of pediatric low-grade glioma (LGG) is 1.3-2.1 cases per 100,000 people, while adult LGG is more common with an estimated 9.1-12.5 cases per 100,000 people (3, 4). Glioblastoma multiforme (Grade IV) is the second most common primary intracranial tumor, and the most common malignant tumor of the central nervous system. GBM accounts for 15.4% of all primary brain tumors and 45.6% of primary malignant brain tumors. Grade I gliomas are essentially benign and respectable (5). A large number of clinical studies have found that the survival rate of LGG patients is low, and many patients have a sharp decline in survival time from tumor deterioration in the later stage (6). The high recurrence and malignancy rates of LGG are detrimental to patients (7, 8). Investigating approaches to maintain the quality of life of LGG patients while prolonging their overall survival (OS) has become a common focus for clinicians and researchers (9–12).

The rapid development of modern bioinformatics and phenotyping has provided great convenience to our research (13–15). Recent work has suggested that the tumor microenvironment (TME) can facilitate the development of tumors (16, 17). The interactions between cancer cells, stromal cells, and recruited immune cells promote the invasion and metastasis of a variety of cancers, as well as cell proliferation, anti-apoptosis signals, and evasion of immune surveillance. This significantly impacts the treatment and prognosis of cancer patients (18, 19). The TME is mainly composed of resident stromal cells and recruited immune cells (20), which affect tumor blood vessel growth and tumor proliferation, respectively. Additionally, tumor-infiltrating immune cells (TICs) in the TME can be used to determine patient prognosis (21), and the related immune genes have an impact on cancer patient survival (22, 23). This correlation has led to improvements in immune-based treatment methods to create immune checkpoint inhibitors and identify prognostic biomarkers for tumor patients (24–26). These studies suggest that the various immune responses of the LGG TME may change the purity of the tumor, thereby affecting the invasive and metastatic abilities of LGG. There is a reported strong connection between LGG and the TME. The higher the stromal and immune scores of LGG display, the lower the purity and higher the aggressiveness of the tumor show. Low glioma purity shows a strong immunophenotype and suggests a poor prognosis (27). Thus, clinicians and basic science researchers are required to identify tumor purities that accurately reflect the LGG heterogeneity and complex role of the microenvironment, which may also help to discover novel biomarkers of LGG.

We selected 509 LGG patients from The Cancer Genome Atlas (TCGA) dataset and calculated the percentage of TICs and tumor purity of each LGG tumor through ESTIMATE and CIBERSORT calculation methods. We also calculated the ratio of immune and matrix components and selected the inter-sample screening in the Gene Expression Omnibus (GEO). LGG genes associated with prognosis were identified and the predictive biomarker *CD3E* was found. The T cell antigen receptor epsilon subunit (*CD3E*) gene is located on chromosome 11q23.3, composed of nine exons, and is associated with autosomal recessive hereditary early-onset immunodeficiency 18 phenotype, which is a severe combined immunodeficiency variant (27). Moreover, *CD3E* is overexpressed in certain solid tumors and is associated with immunity (28, 29). Among the differentially expressed genes (DEGs) produced by comparing immunological and matrix components in LGG samples, we determined that *CD3E* is a potential indicator of TME status changes in LGG. This gene may affect the tumor microenvironment of LGG by regulating T cells, which may be completely different from the tumor microenvironment of other organs outside the skull. The higher the expression of *CD3E* is, the worse the prognosis of LGG patients is.

## Methods

### Data Collection

This study included 509 patients from TCGA (30) database and 487 patients from GEO (30, 31) databases (three datasets, GSE107850 on GPL14951, GSE26576 on GPL6801 and GPL570, GSE20395 on GPL9183, were selected as the second testing cohort for further analysis) two independent testing cohorts. To further improve the clinical value of the study, a total of 100 LGG patients, who underwent surgery in Affiliated Hospital of YouJiang Medical University for Nationalities (AHYMUN, Baise, China) from June 2014 to July 2019, were enrolled in this study. Clinical data of LGG patients that may affect the OS and disease-free survival (DFS) were collected, including age, gender, epilepsy history, capsular invasion Karnofsky score and tumor envelope infiltration.

LGG patients with available RNA sequencing data from the Cancer Genome Atlas (TCGA) database (https://tcga-data.nci.nih.gov/tcga/) were consecutively recruited for the analyses from UCSC Xena (http://xena.ucsc.edu/). UCSC Xena is an online exploration tool for public and private, multi-omic and clinical/phenotype data, and provided level 3 data from TCGA databases. The gene expression profile was measured experimentally using the Illumina HiSeq 2000 RNA Sequencing platform by the University of North Carolina TCGA genome characterization center.

### Tumor Purity Calculation

R software (32) (version 4.0.0) was used to estimate the proportion of TME immune cells and stromal cells in each LGG sample. We use the ssGSEA algorithm to calculate ImmuneScore, StromalScore and ESTIMATEScore (33, 34). The CIBERSORT algorithm is used to calculate the proportion of immune cells in LGG (35).

### Totally 1,068 LGG Patients Included From Online Public and Real-World Cohorts

This study included 509 patients from TCGA database and 487 patients from GEO database (GSE107850, GSE60898, GSE26576) as two independent testing cohorts. To further improve the clinical value of the study, a total of 100 LGG patients, who underwent surgery in Affiliated Hospital of YouJiang Medical University for Nationalities (AHYMUN) from June 2014 to July 2019, were enrolled in this study. Clinical data of LGG patients that may affect the OS and disease-free survival (DFS) were collected, including age, gender, epilepsy history, capsular invasion Karnofsky score and tumor envelope infiltration. Tissue samples were collected during surgery and available from AHYMUN tissue bank. IHC staining of CD3E was performed using a mouse monoclonal anti-CD3E antibody (1:800, ab16669, Abcam, USA) in 100 LGG samples. Positive or negative staining of CD3E protein in a FFPE slide was independently evaluated as previously described (36).

### Screening for Differential Expressed Genes

Using “LIMMA” (37) in R software, standardize the data and perform differential expression analysis. Put the relevant code into R, and analyze the DEGs in LGG samples and normal brain tissue samples through the limma software package. *P* value < 0.05 and Log2FC > 1 was set as the threshold for identifying Clinical-related DEGs.

### Screening for Immune and Stromal Related DEGs

According to the median of the Immune score and the Stromal score, we grouped high and low samples, so as to screen out the TME related genes that highly involved in heterogeneity of tumor immune environment. The 509 LGG samples in the TCGA database were marked as high or low. Use package limma to conduct differential analysis of gene expression, and generate Stromal related DEGs by comparing high and low score samples. Stromal related DEGs (high/low score group) and false discovery rate < 0.05 with a fold change greater than 1 after log2 conversion were considered significant. We calculated the TIC value in all LGG data by the CIBERSORT method, and the samples with *P* < 0.05 can be further analyzed.

### Functional Enrichment Analysis

The protein-protein interaction (PPI) network is constructed from the STRING (38) database. All gene interaction networks were drawn by Cytoscape (version 3.8.0.) (39). We performed gene ontology (GO) enrichment analysis of DEGs through R software, and determined the biological processes (BPs), cell components (CCs) and molecular functions (MFs) of each gene (40). We also performed Kyoto Encyclopedia of Genes and Genomes (KEGG) enrichment analysis to show enrichment for related genes (41). We use GSEA software (vision 4.0.3) to analyze the entire transcriptome of all tumor samples (42), and only genomes with p<0.05 are considered important.

### Immunohistochemistry

Immunohistochemistry streptavidin peroxidase method was used to detect the expression of *CD3E* in tumor, immune and stromal cells from LGG and adjacent normal tissues (43). Immunostaining of CD3E was performed using a rabbit monoclonal anti-CD3E antibody (1:1000, ab237721, Abcam). Positive or negative staining of a certain protein in one FFPE slide was independently assessed by two experienced clinicians, and determined as follows. The LGG samples were scored according to the degree of cell staining intensity and density. Intensity score: 0, cytoplasmic yellow particles; 1, light brown particles; 2, obvious brown particles; 3, a large number of dark brown particles. Density score (according to the percentage of positive cells): 0, 0%, 1, <10%, 2.11%-50%, 3, 51-80%, 4, 80%. The final IHC score is calculated by multiplying the two scores.

### Single-Cell Datasets Processing and Collection

Tumor Immune Single-cell Hub (TISCH, http://tisch.comp-genomics.org/home/) is used to screen for scRNA-seq datasets with detailed cell-type annotation at the single-cell level focusing on tumor microenvironment across different cancers. GSE131928 10X (n = 9, number of cells = 13,553), GSE131928 Smartseq2 (n = 28, number of cells = 7,930), GSE135437 (n = 19, number of cells = 12,559), GSE139448 (n = 3, number of cells = 12,152), GSE141982 (n = 2. Number of cells = 526) and GSE148842 (n = 7, number of cells = 111,397) were enrolled with correlation analysis between CD3E expression and abundance of immune cells infiltrations.

### Cells and Plasmids

Two human glioma cell lines (N9, N33) were cultured in Dulbecco’s modified Eagle medium: nutrient mixture F-12 (DMEM: F12, 01-172-1ACS, Biological Industries) and 10% fetal bovine serum (FBS), 04-001-1A, Bioindustry). CD3E siRNA duplexes were transfected using Lipofectamine 3000 reagent (Invitrogen, USA) according to the manufacturer’s protocol. Cells were used for further analyses after transfection for 48 h. The sequences of siRNA duplexes are listed below: siRNA1#: 5’-UUCUUCAUUACCAUCUUGCCC-3’, siRNA2#: 5’-UAAUACCACCCAUUUCUUCAU-3’.

### Western Blot

After the specified treatment, the cells were harvested and lysed in RIPA buffer and quantified by the bicinchoninic acid assay kit (Pierce, USA). The total protein was separated by sodium dodecyl sulfate polyacrylamide gel electrophoresis (SDS-PAGE) under denaturing conditions and transferred to a nitrocellulose filter (NC) membrane. The membrane was incubated with blocking buffer for 2 hours at room temperature and then with the primary antibody anti-CD3E (1:1000, ab237721, Abcam) overnight at 4°C. Then, the protein was visualized using ECL plus western blotting detection reagents (Biosciences) and detected with an enhanced chemiluminescence kit.

### Cell Counting Kit‐8 Assay

100 microliters of N9 and N33 cell suspension (5 × 10^4^) were added to each well of a 96-well plate, with triple wells in each group. The culture plate was placed in the incubator for pre-culture for 24 hours until the cells stick to the culture dish. Then, we add different concentrations of culture medium to the wells for 24 hours, and add normal and high-sugar medium to the culture plate. After 24 hours, 10 μl CCK-8 solution (#CK04; Dojindo, Japan) was added to each well, and then incubate for 2 hours.

### Transwell Assay

Cell invasion ability was assessed using the Transwell chamber (BD Biosciences). A total of 2 × 10^5^ cells were plated on top of a polycarbonate Transwell filter with 200 mL serum-free medium. The lower compartment is filled with 500 mL of complete medium (1640 + 10% fetal bovine serum). After 24 hours, cells in the upper chamber were removed with cotton swabs, and cells on the underside were fixed with 4% paraformaldehyde for 10 minutes at room temperature. After been washed and air drying, stained cells in four randomly selected fields were photographed and counted under a light microscope (Olympus, Tokyo, Japan).

### Statistical Analysis

In this study, R (Version 3.3.2) and RStudio (Version 1.2) were utilized to perform most data analyses, including Cox regression analyses (44), Kaplan-Meier plots (45), risk plots, PPI network and functional annotations. All tests were two-sided and *p*-value less than 0.05 were taken as significant. The scatter plot was used to represent the differential expression of CD3E in normal and LGG tissues. The primary endpoint, the overall survival of patients who survived specific period of time, which was determined based on the length of time from the date of surgery to the date of death or the date of the last follow-up. Disease-free survival as a secondary endpoint refers to the length of time from the start of curative treatment for which no disease can be found to the date of progression to the date of starting second-line treatment or starting treatment.

## Results

**Figure 1:**
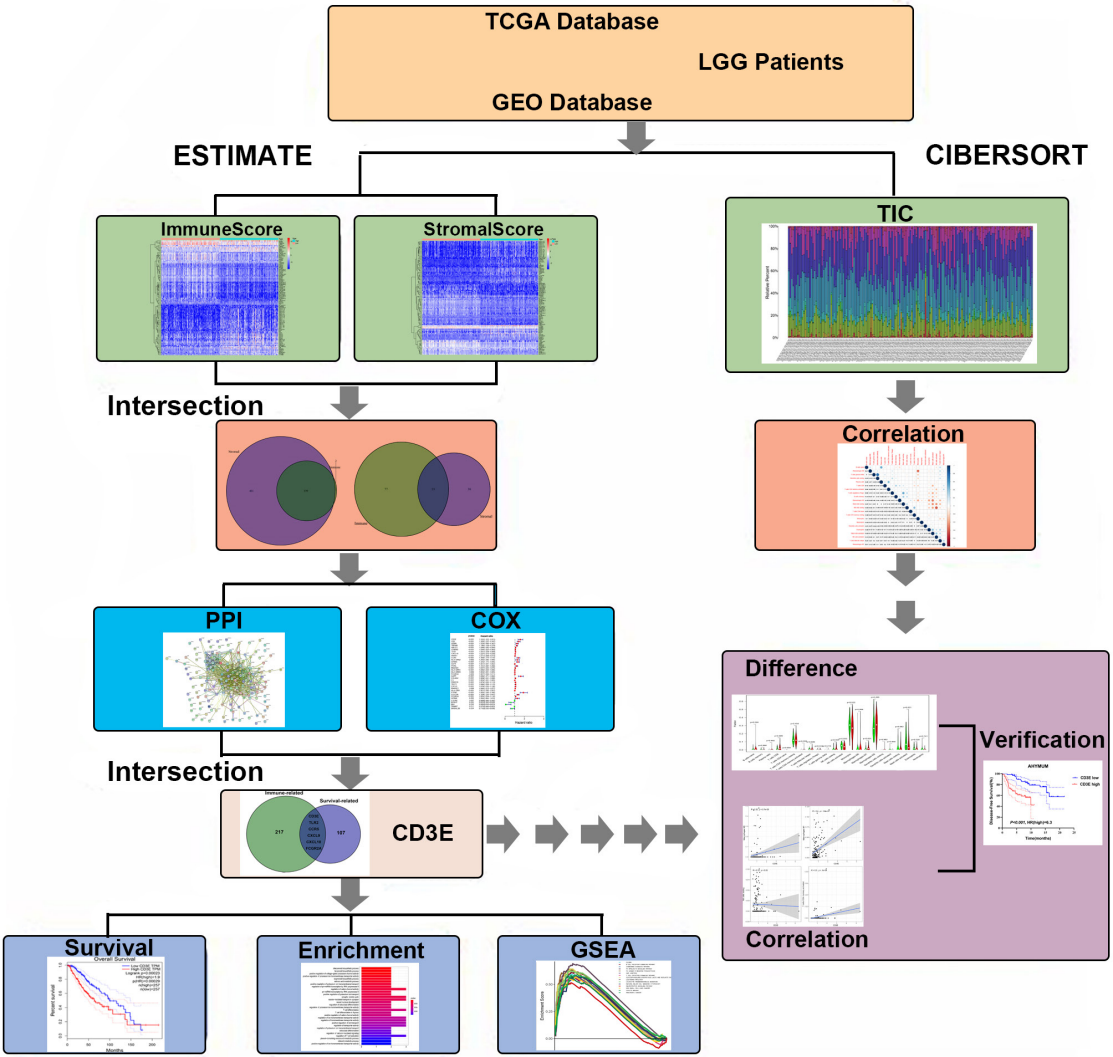
Flowchart of this study. TCGA, the Cancer Genome Atlas; GEO, Gene Expression Omnibus; LGG, low-grade glioma.

As shown in **Figure 1**, this work was conducted in three stages. To estimate the proportion of TICs and tumor purity in LGG samples, transcriptome RNA-seq data from 516 patients were downloaded from TCGA, after which ESTIMATE and CIBERSORT algorithms were performed. DEGs shared by ImmuneScore and StromalScore were used to construct a PPI network. Significant hub genes in the PPI network were evaluated using univariate Cox regression cross-analysis. Additionally, we selected a qualified dataset from the GEO database and conducted a differential analysis to obtain clinical-related DEGs. Then, any associations between the DEGs and LGG patient survival rates were evaluated and screened. Next, *CD3E* was identified and validated as the most relevant gene after combining the two datasets of DEGs. Further studies focused on the impact of *CD3E* on survival, GSEA, and correlation with TICs. Functional annotations of neighboring genes and clinical validation of *CD3E* were performed. Finally, we entered the research conclusions in our own AHYMUN center for clinical cohort study.

### TME-Related Scores Are Related to Survival of LGG Patients

**Figure 2:**
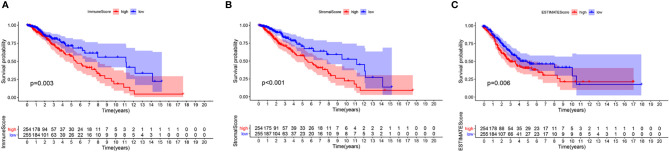
Correlation of scores with the survival of patients with LGG. **(A)** Kaplan-Meier survival analysis for LGG patients grouped into high or low score in ImmuneScore determined by the comparison with the median, *P* = 0.003. **(B)** Kaplan–Meier survival curve for StromalScore, p < 0.001. **(C)** Survival analysis with Kaplan–Meier method for LGG patients grouped by ESTIMATEScore, P = 0.006.

To confirm whether the proportion of cells in the TME and tumor purity can affect the survival time of LGG patients, we calculated ImmuneScore, StromalScore, and ESTIMATEScore and generated a Kaplan-Meier survival curve. The Score was positively associated to the higher the proportion of the corresponding component in the TME. The sum of ImmuneScore and StromalScore is ESTIMATEScore, which also reflects tumor purity. **Figure 2** shows how the TME scores are related to overall survival. ImmuneScore (*P* = 0.003), StromalScore (*P* < 0.001), and ESTIMATEScore (*P* = 0.006) values were positively correlated with OS. These results indicate that the prognosis of LGG patients can be inferred based on the estimated matrix score and help to develop a personalized treatment plan.

### TME-Related Scores Are Related to the Clinical Features of LGG Patients

**Figure 3:**
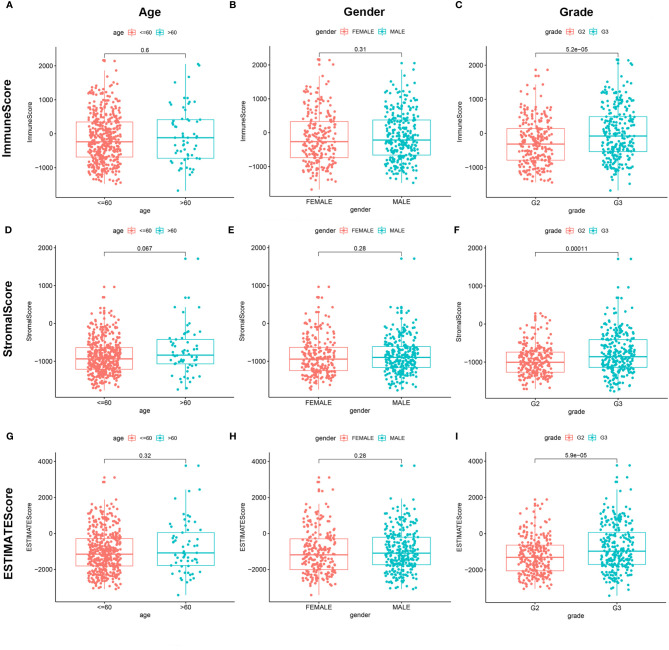
Correlation of ImmuneScore and StromalScore with clinicopathological staging characteristics. **(A, D, G)** Distribution of ImmuneScore, StromalScore, and ESTIMATEScore in age. *P* = 0.6, 0.067, and 0.32. **(B, E, H)** Distribution of three kinds of scores in gender. *P* = 0.31, 0.28, 0.28. **(C, F, I)** Distribution of scores in grade. *P* < 0.001.

We combined the corresponding clinical information of TCGA LGG patients with the above calculated scores to determine whether the LGG TME and tumor purity are related to the patient’s clinical characteristics. ImmuneScore positively correlated with high grade LGG (**Figures 3A–C**, *P <* 0.001), StromalScore also positively correlated with high grade LGG (**Figures 3D–F**, *P <* 0.001), and ESTIMATEScore accompanied with high grade LGG (**Figures 3G–I**, *P <* 0.001). These results indicate that tumor purity and the ESTIMATE scores in the TME are related to the deterioration of LGG. The higher the ESTIMATE scores in the TME, the lower the purity of the tumor and the worse the prognosis of LGG patients.

### Enrichment Analyses of Stromal Related DEGs

**Figure 4:**
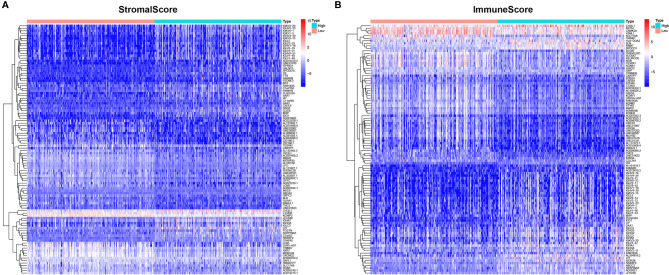
Heatmaps, Venn plots for DEGs. **(A)** Heatmap for DEGs generated by comparison of the high score group vs. the low score group in ImmuneScore. Row name of heatmap is the gene name, and column name is the ID of samples which not shown in plot. Differentially expressed genes were determined by Wilcoxon rank sum test with q = 0.05 and fold-change > 1 after log2 transformation as the significance threshold. **(B)** Heatmap for DEGs in StromalScore, similar with **(A)**.

**Figure 5:**
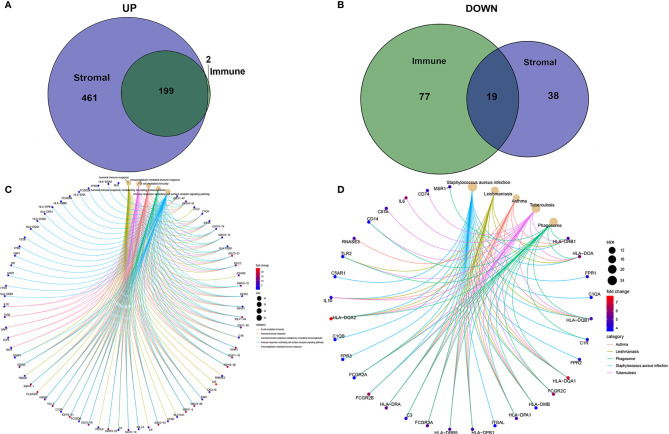
Up-regulated and down-regulated and enrichment analysis of GO and KEGG. **(A, B)** Venn plots showing common up-regulated and down-regulated DEGs shared by ImmuneScore and StromalScore. **(C, D)** GO and KEGG enrichment analysis for DEGs.

To determine the exact changes in the genetic profiles of immune and matrix components in the TME, we used the two packages “limma” (46) and “pheatmap” (47, 48) for analysis, we set the filter conditions to “fdrFilter = 0.05, logFCfilter = 1.5”, by reading the expression input file, deleting the normal sample, reading the score file, according to the score The median value groups the samples, performs difference analysis, and outputs the differences of all genes, and then screens out genes that affect survival. We compared high- and low-scoring samples based on the median value (**Figure 4**). We obtained 518 DEGs from StromalScore, which contained 461 upregulated genes and 57 downregulated genes (**Figure 4A**). We also obtained 297 DEGs through ImmuneScore, with 201 upregulated genes and 96 downregulated genes (**Figure 4B**). Through a Venn diagram, we determined that 199 upregulated genes with high scores and 19 downregulated genes with low scores were contained in both ImmuneScore and StromalScore (**Figures 5A, B**). These 223 stromal related DEGs may play a decisive role in the LGG TME. Through GO enrichment and KEGG analyses, we found that the biological functions of these genes are mainly related to immunity (**Figures 5C, D**).

### Identification of Key Stromal Related Genes

**Figure 6:**
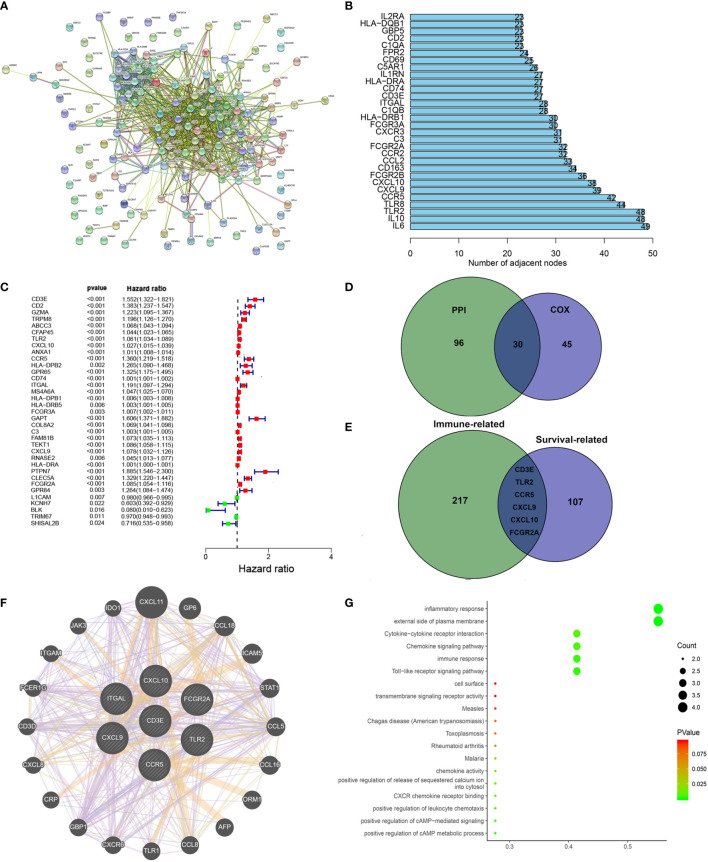
Protein–protein interaction network and univariate COX. **(A)** Interaction network constructed with String. **(B)** The top 30 genes ordered by the number of nodes. **(C)** Univariate COX regression analysis with DEGs was done, listing the top significant factors with *P* < 0.001. **(D)** Venn plot showing the common factors shared by nodes in PPI and top significant factors in univariate COX. **(E)** Venn plot showing the common factors shared by nodes in Stromal related DEGs and Clinical-related DEGs. **(F)** Interaction network constructed with 7 genes. **(G)** GO and KEGG pathway analyses on 7 genes.

To further study the underlying mechanisms of the abovementioned genes and determine which were most crucial, we generated a PPI network diagram through String. The interactions between the genes are shown in **Figure 6A**. We selected the top 30 genes ranked by the number of nodes and plotted them as a bar graph (**Figure 6B**). We performed univariate Cox regression analysis on stromal related DEGs and LGG patient survival to determine which genes are most high risk for LGG patients and which are low risk (**Figure 6C**). Finally, we combined the main nodes in the PPI diagram and the top 75 genes ranked by *P* value to analyze them, and obtained 30 intersecting genes. (**Figure 6D**). These genes are significantly related to the prognosis of LGG.

### Filtering Clinical-Related DEGs to Identify a Target Gene

We used the R language package “limma” (46) to screen the genes that affect survival in three GEO sets (GSE107850, GSE60898, GSE26576). We screened 114 clinical-related DEGs (*P <* 0.001) that were significantly related to survival from a group of 13,299 genes and compared them with the previous stromal related DEGs to obtain seven genes: *CD3E*, *TLR2*, *CCR5*, *CXCL9*, *CXCL10*, *FCGR2A*, and *ITGAL* (**Figure 6E**). We mapped the PPI network for these seven genes (**Figure 6F**). 78.89% of terms were in co-expression (lavender line), 7.65% of terms were shared protein domains (yellow line), 7.11% of terms were in co-localization (deep blue line), and 7.11% of terms were predicted (khaki line). We also performed GO and KEGG pathway analyses on these seven genes, finding that the genes were related to immune diseases and the inflammatory response (**Figure 6G**).

Next, in order to reduce system bias and select multiple cohorts with large samples to increase the rigor of the research, we also screened the clinically relevant genes in the GEO database. We selected a suitable data set from the GEO database for clinical analysis (GSE107850, GSE60898, GSE26576), comparing it with the immune-related genes, based on the hazard ratio (HR) value of each gene and the survival-related P value, we targeted CD3E for further study.

**Figure 7:**
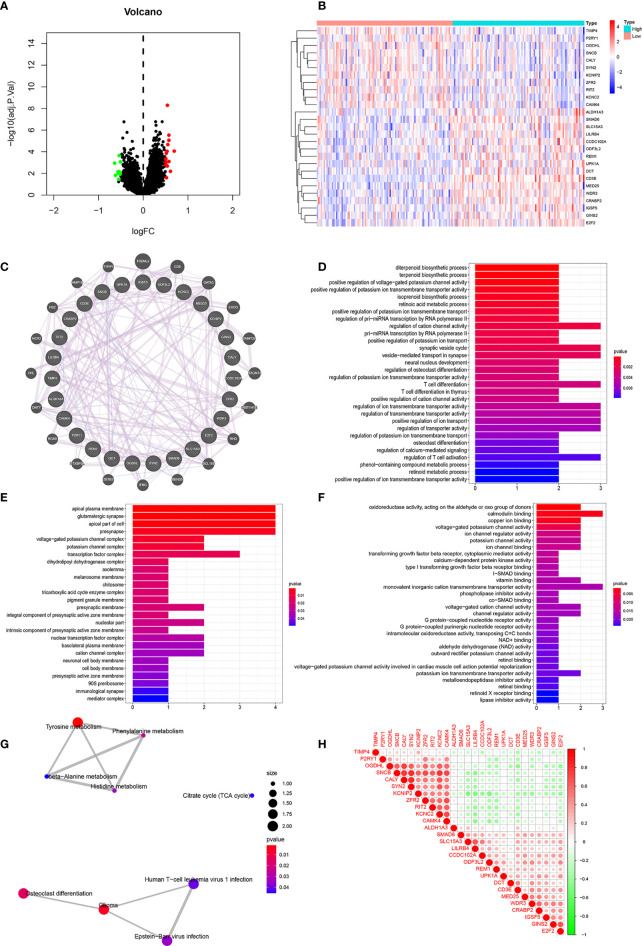
Correlation Analyses of Clinical-Related DEGs **(A)** The volcano map of Clinical-Related DEGs. **(B)** The heat map of Clinical-Related DEGs. **(C)** PPI of Clinical-Related DEGs. co-expression (lavender line), co-localization (deep blue line). **(D)** The enrichments of biological processes of DEGs. **(E)** The enrichments of cellular components of DEGs. **(F)** The enrichments of molecular functions of DEGs. **(G)** The enrichments in KEGG pathway of DEGs. **(H)** The 20 most significantly up-regulated genes and the 20 most significantly down-regulated genes with *CD3E*. Red color was for positive correlation, and green color represented a negative correlation. The deeper the color indicated the greater the relevance.

We divided the dataset into high and low expression groups according to the median *CD3E* expression value and screened using “log fold change = 0.5, and *P <* 0.05”. A total of 114 related differential genes were obtained. The 15 genes with the most significant up-regulation and the 11 genes with the most significant down-regulation were selected for further analysis (**Table S1**), which were visualized with a volcano map (**Figure 7A**) and heat map (**Figure 7B**).

### Correlation Analyses of Clinical-Related DEGs and Functional Enrichment Analysis of CD3E in LGG

As illustrated in **Figure 7C**, gene-gene interactions between clinical-related DEGs were analyzed. 95.20% of terms were in co-expression (lavender line) and 4.80% terms were in co-localization (deep blue line). We then conducted a biological function enrichment analysis of clinical-related DEGs. The results showed that enrichments of biological processes included positive regulation of voltage-gated potassium channel activity, positive regulation of potassium ion transmembrane transporter activity, and regulation of pri-miRNA transcription by RNA polymerase II (pol II) (**Figure 7D**); enrichments of cellular components included ion glutamatergic synapse, apical plasma membrane, and apical part of cell (**Figure 7E**); enrichments of molecular functions included oxidoreductase activity, calmodulin binding, and copper ion binding (**Figure 7F**). Enrichments in KEGG pathway analysis were glioma, tyrosine metabolism, and citrate cycle (**Figure 7G**).

We correlated the 20 most significantly up-regulated genes and the 20 most significantly down-regulated genes with *CD3E*. As shown in **Figure 7H**, red represents a positive correlation and green represents a negative correlation. The deeper of the color indicated the greater the relevance. *CD3E* was positively correlated with *LILRB4*, *UPK1A*, and *REM1*, and negatively correlated with *RIT2*, *OGDHL*, and *KCNC2* (**Figure 7H**).

Besides, as shown in **Figures S1A, B**, we identified 866 up-regulated genes and 256 down-regulated genes based on top 25% high (G1) and low (G2) CD3E expression in total 256 LGG patients from TCGA using Limma R package with |LogFC| > 2, *P* < 0.05. GO and KEGG enrichment could effectively suggest gene functions and associated high-level genome functional information in **Figures S1C, F**. In addition of this role of signal transduction in T-cell activation and proliferation, CD3E plays an essential role in correct T-cell development, neutrophil activation involved in immune responses, cell adhesion molecules and extracellular matrix organization, thus reshaping suppressive TME and promoting malignant behaviors of LGG.

### *CD3E* Expression Is Negatively Related to LGG Patient Survival

**Figure 8:**
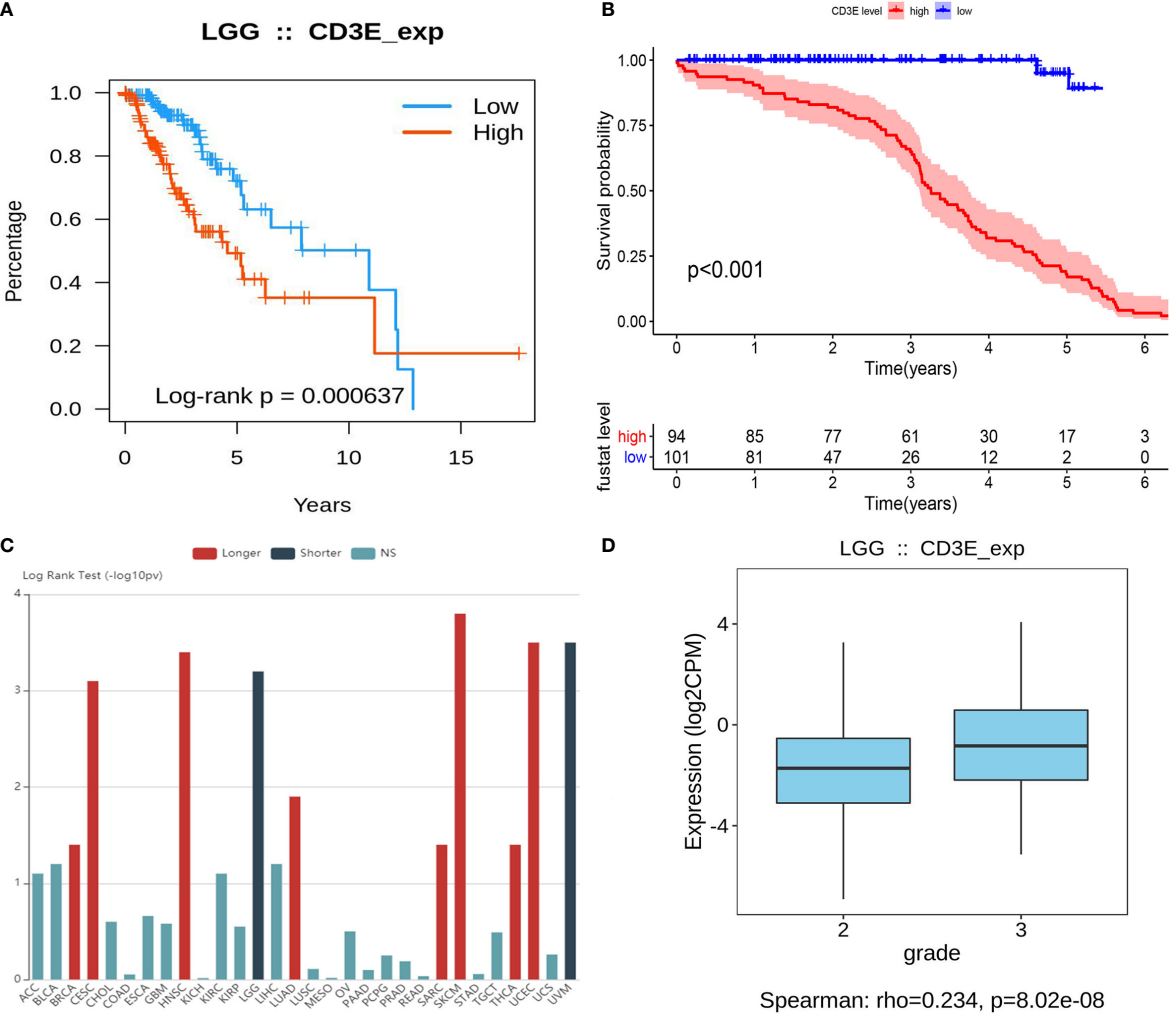
Relationship between *CD3E* expression and survival of LGG patients **(A)** Relationship between *CD3E* expression and survival of LGG patients in the TCGA database (*P* = 0.000637). **(B)** Relationship between *CD3E* expression and survival of LGG patients in the GSE database (*P* < 0.001). **(C)** CD3E might have completely opposite prognostic effect of CD3E in gliomas than that of most other tumors. **(D)** The relationship between *CD3E* expression and survival of LGG patients.

*CD3E* is an epsilon subunit of the T cell antigen receptor. According to the *CD3E* expression median value, all LGG samples were divided into *CD3E* high and low expression groups. Analysis of the TCGA data (*P* = 0.000637; **Figure 8A**) and GEO data (*P <* 0.001; **Figure 8B**) suggested that the survival rate of LGG patients with high *CD3E* expression was lower than those with low *CD3E* expression. Interestingly, after a literature review and pan-cancer statistical tests (16, 49), we found that *CD3E* may have an opposite prognostic effect in gliomas than in most other tumors (**Figure 8C**). Moreover, it is only in the two head tumors of uveal melanoma and LGG that the higher the expression is, the worse the prognosis is (**Figure S2**). Finally, we studied the difference in *CD3E* expression between Grade II and Grade III patients in the TCGA cohort. We found that patients with higher grades had higher expression levels of *CD3E* and worse prognosis in the clinic (**Figure 8D**).

At the same time, we conducted a subgroup analysis of different clinical characteristics on clinical data to eliminate clinical bias. We found that the effect of CD3E is still the same in LGG patients with different clinical characteristics (**Figure S3**). Then, we explored differential *CD3E* expression based on the histological subtypes of LGG. Significantly elevated *CD3E* expression was found in astrocytoma samples (*n* = 194) compared with oligoastrocytoma samples (*n* = 130, *P* = 6.43 × 10^-4^) or oligodendroglioma samples (*n* = 130, *P* = 6.4187 × 10^-4^) (**Figure S4**).

### Correlation of *CD3E* With the Proportion of TICs

**Figure 9:**
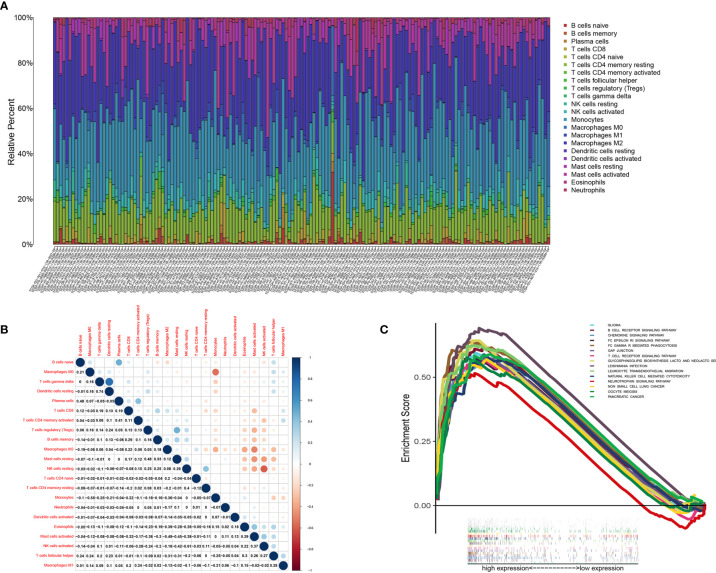
TIC profile in tumor samples and correlation analysis. **(A)** Barplot showing the proportion of 22 kinds of TICs in LGG tumor samples. Column names of plot were sample ID. **(B)** Heatmap showing the correlation between 22 kinds of TICs and numeric in each tiny box indicating the p value of correlation between two kinds of cells. The shade of each tiny color box represented corresponding correlation value between two cells, and Pearson coefficient was used for significance test. **(C)** GSEA for samples with high *CD3E* expression.

We used the CIBERSORT algorithm to analyze the proportion of TICs for 22 immune cells in LGG to further study the correlation between *CD3E* and the immune microenvironment of LGG. (**Figures 9A, B**). Considering that *CD3E* expression is negatively correlated with the survival rate of LGG patients, we performed GSEA analysis on the high expression group. We found that the genes in the *CD3E* high expression group mainly participated in stromal related activities, such as the B cell receptor signaling pathway, chemokine signaling pathway, and T cell receiver signaling pathway (**Figure 9C**). Furthermore, *CD3E* was positively related to glioma and immune cell response. These results suggest that *CD3E* may be a potential indicator of TME status for LGG.

**Figure 10:**
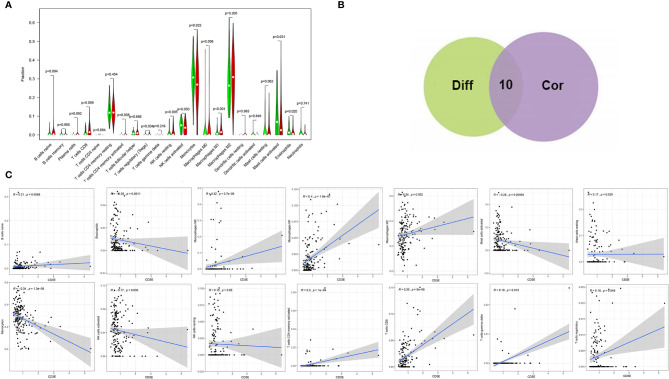
Correlation of TICs proportion with *CD3E* expression. **(A)** Violin plot showed the ratio differentiation of 22 kinds of immune cells between LGG tumor samples with low or high *CD3E* expression relative to the median of *CD3E* expression level, and Wilcoxon rank sum was used for the significance test. **(B)** Venn plot displayed ten kinds of TICs correlated with *CD3E* expression codetermined by difference and correlation tests displayed in violin and scatter plots, respectively. **(C)** Scatter plot showed the correlation of 14 kinds of TICs proportion with the *CD3E* expression (*P* < 0.05). The red line in each plot was fitted linear model indicating the proportion tropism of the immune cell along with *CD3E* expression, and Pearson coefficient was used for the correlation test.

We found that the expression of *CD3E* is related to 10 groups of TICs in LGG (**Figure 10**). Seven kinds of TICs were positively correlated with *CD3E* expression, including M0 macrophages, M1 macrophages, resting mast cells, resting NK cells, CD4^+^ memory activated T cells, CD8^+^ T cells, and regulatory T cells. Three kinds of TICs were negatively correlated with *CD3E* expression, including eosinophils, monocytes, and activated NK cells. Then, we calculated the relationship between the abundance of tumor infiltrating lymphocytes and the expression, copy number, methylation, or mutation of *CD3E* in LGG (**Figure S5**). These results suggest that *CD3E* is related to the immune activity of the TME, thereby affecting the tumor purity of LGG.

Next, we aimed to investigate predictive role of CD3E expression in predicting responses to immune checkpoint inhibitors of LGG using Tumor Immune Dysfunction and Exclusion (TIDE) algorithm. Interestingly, we found that TIDE score was significantly higher in CD3E^high^ group compared with CD3E^low^ group in 255 LGG patients (*P* = 0.001), suggesting poor prognosis of LGG patients with high CD3E expression and the poor tolerance of immune checkpoint inhibitor therapy (**Figure S6**).

### Single Cell Analysis of CD3E in Brain Tumors

**Figure 11:**
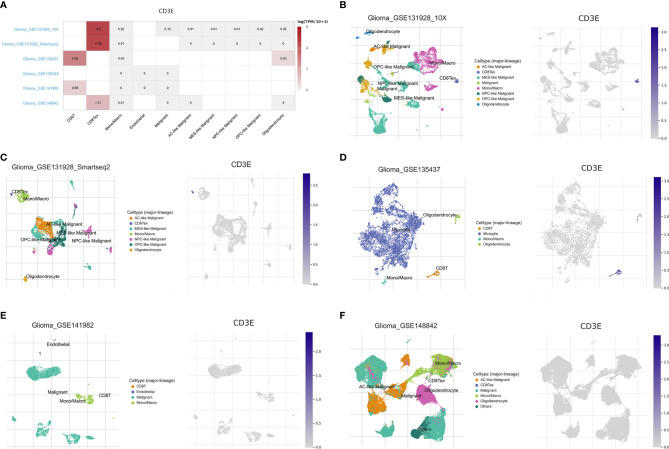
Single cell analysis of CD3E in LGG. Six single cell sequencing data sets from geo analysis were used to analyze the expression of cd3e in CD8 + T cells. **(A)** We quantitatively calculated the positioning and binding of CD3E on various immune cells across the dataset using a heatmap. **(B–F)** Five single-cell RNA-seq datasets were enrolled to determine the location of CD3E in different cell.

To further explore the mechanism by which CD3E promotes tumor immune evasion in brain tissue and LGG, we performed complex bioinformatics work including functional enrichment and GSEA analyses. The results suggested that CD3E is more likely to participate in T cell-regulated immune deficiency as one of its important roles in the formation of the TCR. Next, we enrolled six glioma single-cell sequencing datasets from GEO analysis (GSE131928 10X, GSE131928 Smartseq2, GSE135437, GSE139448, GSE141982, and GSE148842), which suggested significantly elevated *CD3E* expression in CD8^+^ T cells, especially the exhaustive T cells. Therefore, we hypothesize that CD3E possibly contributes to an immune evasion mechanism in brain tumors by leading to T cell dysfunction (**Figure 11**).

### Clinicopathological Features Related to *CD3E* Expression

**Figure 12:**
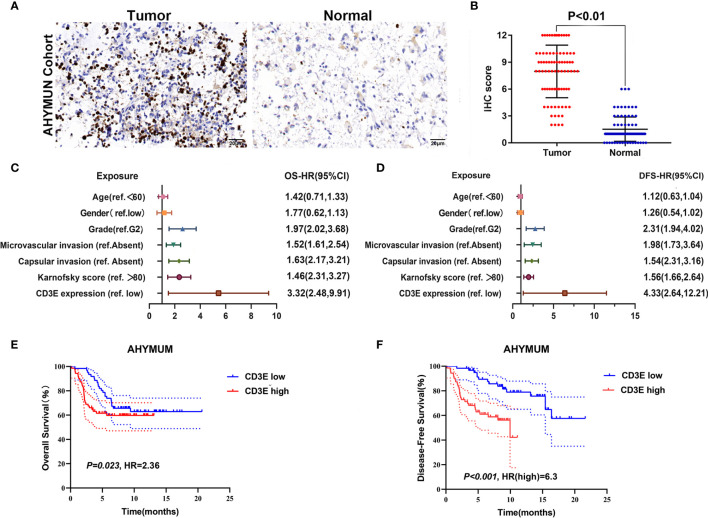
The relationship between *CD3E* gene and LGG prognosis was further verified. **(A)** IHC on collected LGG tissue. **(B)** The scatter plot of the IHC scores (*P <* 0.01). **(C, D)** Forest plots were used to visualize the univariate Cox regression analysis of OS and DFS in the AHYMUM cohorts. **(E, F)** Survival curves showed that LGG patients with elevated *CD3E* expression levels in the AHYMUN cohort showed poorer OS (*P* = 0.023) and poorer DFS (*P <* 0.001).

To verify *CD3E* expression in LGG, we performed immunohistochemistry (IHC) (**Figures 12A, B**). The scatter plot of the IHC scores revealed that *CD3E* expression increased in LGG tissues in the AHYMUN cohort (*P <* 0.01). In **Table 1**, we show that higher *CD3E* expression correlates with patient age (*P* = 0.027), grade (*P <* 0.001), microvascular invasion (*P* = 0.009), history of epilepsy (*P <* 0.001), and Karnofsky score (*P* = 0.002). We believe this indicates that the higher the expression of *CD3E* in patients, the worse the prognosis.

**Table 1:** Clinicopathological characteristics in relation to CD3E expression level in AHYMUM cohort.

Characteristics	AHYMUN cohort	CD3E expression		χ^2^	*P*
	(N=100)	Low IHC score	High IHC score		
		(N = 50)	(N = 50)		
N (%)					
Age				4.889	**0.027**
<60 years	55 (0.55)	33 (0.60)	22 (0.40)		
≥60 years	45 (0.45)	17 (0.38)	28 (0.72)		
Gender				0.271	0.603
Male	82 (0.82)	40 (0.49)	42 (0.51)		
Female	18 (0.18)	10 (0.56)	8 (0.44)		
Grade				14.924	**<0.001**
G2	69 (0.69)	39 (0.57)	30 (0.43)		
G3	31 (0.31)	11 (0.35)	20 (0.65)		
Seizure history				12.148	**<0.001**
yes	61 (0.61)	39 (0.64)	22 (0.36)		
no	39 (0.39)	11 (0.28)	28 (0.72)		
Microvascular invasion				6.828	**0.009**
Absent	55 (0.55)	34 (0.62)	21 (0.38)		
Present	45 (0.45)	16 (0.36)	29 (0.64)		
Capsular invasion				1.961	0.161
Absent	51 (0.51)	29 (0.57)	22 (0.43)		
Present	49 (0.49)	21 (0.43)	28 (0.57)		
Karnofsky score				9.180	**0.002**
≥80	61 (0.61)	36 (0.59)	21 (0.41)		
<80	39 (0.39)	14 (0.36)	29 (0.64)		

IHC, immunohistochemistry; AHYMUN, Affiliated Hospital of YouJiang Medical University for Nationalities.

P value less than 0.05 was considered as statistical significance and marked in bold.

### Cox Regression Analysis

We used univariate Cox regression analysis to demonstrate the relationship between *CD3E* and AHYMUN patients and found that *CD3E* is not significantly related to age and gender (**Figure 12C**). In the multivariate model, we also found that patients in the high expression group had worse OS (HR = 3.22; *P* = 0.001). Moreover, in the AHNTU cohort, the microvascular invasion (HR = 1.52; *P* = 0.024), the presence of capsular infiltration (HR = 1.63; *P* = 0.016), and the Karnofsky scores (ref < 80) (HR = 1.46; *P* = 0.023) were associated with low OS (**Table 2**).

**Table 2:** Multivariate Cox regression analysis of DFS and OS in AHYMUM cohorts.

Covariates	OS			DFS		
	HR	95% CI	*P* value	HR	95% CI	*P* value
Grade (ref. G2)	1.97	2.25-3.68	**0.043**	2.31	1.94-4.02	**0.037**
Microvascular invasion (ref. Absent)	1.52	1.61-2.54	**0.024**	1.98	1.73-3.64	**0.031**
Capsular invasion (ref. Absent)	1.63	2.17-3.21	**0.016**	1.54	2.31-3.16	**0.017**
Karnofsky score (ref. >80)	1.46	2.31-3.27	**0.023**	1.56	1.66-2.64	**0.044**
CD3E expression (ref. low)	3.32	2.48-9.91	**0.001**	4.33	2.64-12.21	**<0.001**

DFS, disease-free survival; OS, overall survival. P value less than 0.05 was considered as statistical significance and marked in bold.

We found that the patient’s gender and epilepsy history were not related to DFS (**Figure 12D**). We found through Cox analysis that the high expression of the *CD3E* gene caused a significant decrease in OS (HR = 4.33; *P <* 0.001) (**Table S1**). Grade, capsular infiltration, microvascular invasion, and Karnofsky scores were related to OS (*P <* 0.05). As seen in **Figures 12E, F**, the higher the *CD3E* expression level, the lower the OS and DFS of LGG patients.

### Down-Regulation of CD3E Inhibits Cell Proliferation and Invasion Abilities in N9 and N33 Cells

**Figure 13:**
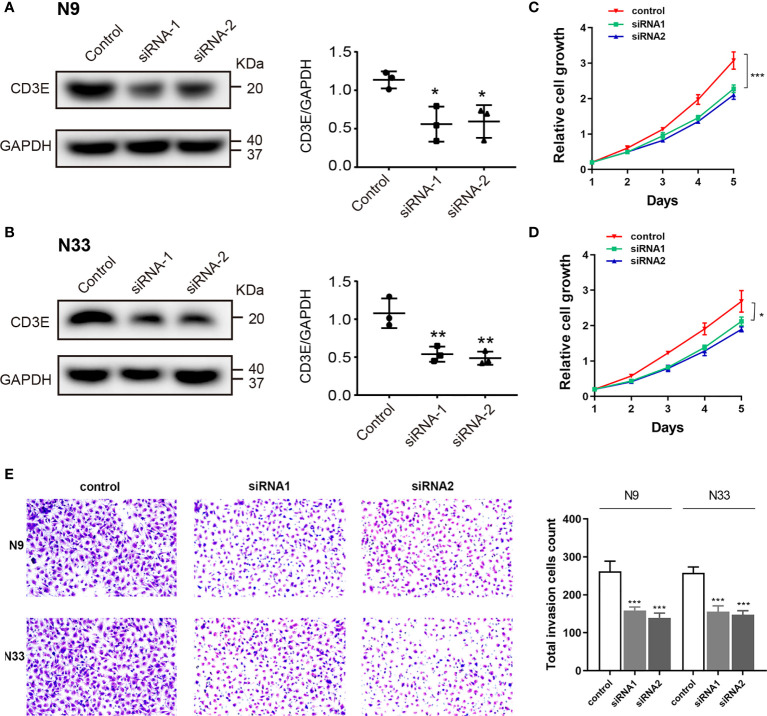
Down-regulation of CD3E inhibits cell proliferation and invasion abilities in LGG cells. **(A, B)** Western blot showed that CD3E protein expression was markedly decreased after siRNA-CD3E transfection. **(C, D)** CCK8 assay showed that the decreased CD3E expression significantly inhibited cell proliferation in N9 and N33 cells. **(E)** Transwell assay showed that when expression of CD3E was inhibited, the invasion ability of N9 and N33 cell lines was significantly reduced.

To explore biological malignancy of CD3E in LGG, we used siRNA methods to restrain the expression of CD3E. Western blot showed that CD3E protein expression was markedly decreased after siRNA-CD3E transfection, compared with the negative control group (**Figures 13A, B**). CCK8 assay showed that the decreased CD3E expression significantly inhibited cell proliferation in N9 and N33 cells (**Figures 13C–E**). Still, we found that when expression of CD3E was inhibited, the invasion ability of N9 and N33 cell lines was significantly reduced compared with normal genitive control group (**Figure 13E**). Taken together, down-regulated CD3E expression significantly restrained LGG cells proliferation and invasion capacities, thus may reducing the malignant biological behaviors and aggressive progression of LGG.

## Discussion

In this study, we first screened the immune genes related to the TME in LGG patients from the TCGA database. Next, we screened genes related to the prognosis of LGG patients from GEO. After combining the above genes, we determined *CD3E* to be the main target gene. Then, we conducted a series of bioinformatics analyses and verified the research results at our own center. We found that *CD3E* may be an indicator gene of the TME status of LGG patients and, by affecting the TME of LGG, can thereby change the tumor purity and affect the prognosis of patients.

The combination of the cancer cell genotype, its gene expression pattern, and the influence of the TME determines the tumor’s adaptability, evolution, and resistance to treatment (50). In recent years, studies using TCGA and GSE have mapped the genetic picture and overall expression status of numerous tumors, identified driver mutations, and defined tumor subtypes based on specific transcription profiles (51, 52).

LGG is a common brain tumor, and the prognosis of patients with WHO grade II and III is normally poor (53). However, surgery, radiation therapy, or chemotherapy (usually using temozolomide) often cannot improve the prognosis and survival of patients (54, 55). The reasons for the lack of progress include the growth of invasive tumors in basic organs, which limits the utility of local therapies. Additionally, the protection of tumor cells by the blood-brain barrier limits the drug concentration, while the blood-tumor barrier protects tumor cells (56). When pursuing immune-based glioblastoma treatment methods, the unique immune environment of the central nervous system needs to be considered (57–59). Therefore, we need to study novel LGG immunotherapy candidates. Here, we began with the transcriptional analysis of LGG data in TCGA and found that the decreased expression of *CD3E* is closely related to poor prognosis of patients. Therefore, *CD3E* is a potential prognostic indicator and treatment target in LGG patients.

Yoshihara et al. developed an algorithm for evaluating tumor purity (60), using gene expression data to evaluate the presence of antigen cells and the penetration of immune cells in tumor samples. The evaluation algorithm proved to be a robust tumor fine prediction algorithm. Previous studies have shown that low tumor purity is associated with poor prognosis in colon cancer, gastric cancer and glioma (27, 61). However, there are few studies on specific genes that affect tumor purity and thus affect LGG. Our research shows that the purity of tumors affected by CD3E plays an important role in predicting the prognosis and genomic status of LGG. The higher the expression of CD3E, the lower the purity of LGG tumors, which is associated with enhanced immune escape and poor prognosis, suggesting that patients with low-purity LGG may benefit more from immunotherapy. In order to better understand TME and make better clinical decisions, further research on tumor purity is needed.

*CD3E* encodes the polypeptide CD3-ϵ, which together with the CD3-γ, -δ and -ZETA and T-cell receptor α/β and γ/δ T cell receptor heterodimer -CD3 complex. The complex plays an important role in coupling antigen recognition to several intracellular signal transduction pathways, so defects in *CD3E* can lead to immunodeficiency (62). CD3E role as a biological component that is functionally important for T cell receptor signaling for proper immunity. This is why the molecule appeared to be increased and as they proposed that would relate to a poorer prognosis. In fact, the more T cells in the tissue would mean that T cell immunity occurs there to act against tumor cells as ones would expect. However, there are many T cell subsets most of which have CD3E as the TCR/CD3 complex component, yet they perform different function ranging from protection (e.g., CD4 and CD8 T cells against viruses and tumors), autoimmune (self-reactive T cells), to those that suppress other T cells (e.g., regulatory T cells). CD3E is a part of the TCR-CD3 complex on the surface of T lymphocytes, and its basic immune function plays a vital role in the adaptive immune response. When antigen-presenting cells activate T cell receptors, TCR-mediated signals are transmitted across the cell membrane through the CD3 chain CD3D, CD3E, CD3G, and CD3Z, thereby activating downstream signaling pathways. In addition to the role of signal transduction in T cell activation, CD3E also plays a vital role in correct T cell development. The TCR-CD3 complex assembly is initiated by forming two heterodimers CD3D/CD3E and CD3G/CD3E. It also participates in the internalization of the TCR-CD3 complex and the down-regulation of the cell surface through the endocytosis sequence present in the cytoplasmic region of CD3E (49, 63). CD3E also participates in proper T cell development. TCR-CD3 complex assembly is initiated by the formation of two heterodimers: CD3D/CD3E and CD3G/CD3E. Additionally, CD3E participates in the internalization of TCR-CD3 complexes and cell surface down-regulation by endocytic sequences present in the cytoplasmic region of CD3E (49, 63, 64). The relationship between the abundance of tumor infiltrating lymphocytes and the expression, copy number, methylation, or mutation of *CD3E* in LGG is shown in **Figure S1**.

In LGG patients, the higher the expression of *CD3E* signified the worse the patient’s survival. It may be attributed to immune cells with high *CD3E* expression promoting anti-tumor immunity, except regulatory T cells. Similarly, CD3E acts as a T cell receptor. Its high expression in many cancers indicates better clinical results (longer survival), with the lone exception of LGG (65). This may be related to the cause of LGG and the immune environment of the brain, or it may be due to the interconnection between isocitrate dehydrogenase and the TME (66–68). In CD3E knockdown experiments, we found down-regulated CD3E expression significantly restrained LGG cells proliferation and invasion capacities, thereby further reducing the malignant biological behaviors and aggressive progression of LGG, which may be closely related to the functional involvement of CD3E in TME of LGG. Studies have shown that combining CD3E antibodies with antibodies that bind to mutant epidermal growth factor receptor variant III can effectively treat mice with gliomas (69). Therefore, *CD3E* may play a dual role in tumors, either promoting survival or inducing apoptosis. In our Western blot and CCK8 experiments, we found that the higher the expression of CD3E represented the higher the invasion of tumor cells. This is one of the reasons why the higher the expression of CD3E, the worse the survival of LGG patients.

In addition, in the TME of glioma, the proliferation of malignant cells is enhanced, the pool of undifferentiated glioma cells increases, and macrophage expression exceeds microglial expression (65–68). Still, it is an interesting question that *CD3E* may have a completely opposite prognostic effect in gliomas than that in most other tumors. In this study, *CD3E* was selected because it had the most significant prognostic value (HR=1.552, *P*<0.001) of LGG. DEGs screened according to *CD3E* expression were mainly involved in stromal related activities. Additionally, significantly increased *CD3E* expression was found in 100 LGG samples from a validation cohort compared with adjacent normal brain tissues. High *CD3E* expression could serve as an independent prognostic indicator for OS and DFS of LGG. CD3E normally plays an important role in the formation of the TCR and participates in multiple signaling pathways in T cell-regulated immune deficiency. After a literature review and pan-cancer statistical tests, we found that *CD3E* may have a completely opposite prognostic effect in gliomas than in most other tumors (**Figure 8C**), except for Uveal Melanoma (**Figure S2**) and LGG. In our research, we found that CD3E is highly expressed in T cells. Through bioinformatics and immunohistochemistry studies, we found that CD3E is also highly expressed in LGG. Therefore, we studied the expression of CD3E in pan-cancer cell lines (**Figure S7**), and we found that the expression of CD3E in all tumors is not the highest in gliomas. However, in the above studies, we found that the higher the expression of CD3E, the worse the prognosis of LGG, which is completely opposite to tumors such as liver cancer and breast cancer. We considered that CD3E plays an active role in most TMEs and passed It binds to T cell surface receptors in the form of a complex to regulate T cell-mediated anti-tumor immune evasion. The immune system is usually limited to the brain. The activation of various immune cells in LGG makes TME different from other solid tumors. Therefore, we hypothesized that CD3E, as one of the main regulatory elements of the LGG immune microenvironment, may play an important role in LGG immune evasion and the shaping of the immunosuppressive microenvironment. In the previous bioinformatics analysis, we found through single cell analysis of brain tumors that the expression of CD3E is particularly prominent in CD8^+^ T cells. Therefore, we hypothesized that CD3E may promote the immune evasion mechanism of brain tumors by causing T cell dysfunction in the immune cell population. In subsequent experiments, we found that the higher the expression of CD3E in tumor cells, the stronger the invasion ability of LGG. We know that the cells and molecules in TME are in a process of dynamic changes at any time. Stromal cells and immune cells jointly promote the proliferation, apoptosis, metastasis and immune escape of cancer cells (70); while tumor invasion and infiltration are often time-sensitive, influencing TME all the time. Therefore, we made an audacious hypothesis that the reason why CD3E can be used as an independent molecular marker to test the prognosis of LGG patients is because it affects both immune cells and tumor cells. It can be said that in LGG, CD3E is the key gene for tumor cells and TME to influence each other, and it is the bridge between the two. Further studies would focus on the underlying mechanism of CD3E in immune microenvironment of LGG.

Our GSEA results also suggested that high *CD3E* expression enriched stromal related signaling pathways, such as B/T cell receptor signaling pathways and chemokine signaling pathways. These results indicate that *CD3E* may be involved in the transition of the TME from immune-based to metabolic-based. An increasing number of studies show that *CD3E* is related to tumor treatment (71–73). Our research also found that the balance between tumor pathways, sugar metabolism, and lactic acid formation can affect the immune status of LGG. Therefore, we suspect that in the development of LGG, the up-regulation of *CD3E* promotes the decline of tumor purity. Simultaneously, the transition of the TME from immune-based to metabolic-based further promotes the deterioration of LGG.

We also found that positive regulation of voltage-gated potassium channel activity is related to LGG. MicroRNAs (miRNAs) can reportedly promote the development of invasive nonfunctional pituitary adenomas (74, 75). Current knowledge suggests that voltage-gated potassium channels play a fundamental role in the generation and transmission of action potential (76), but their role in tumors has not been deeply studied. Whether genes can affect the tumor immune microenvironment through action potential is an area of further research. We found that positive regulation of potassium ion transmembrane transporter activity is related to LGG as well, so we can confirm that potassium ions play an important role in LGG. Previous studies have found that DNA methylation promotes the invasion and development of osteosarcoma through potassium ion transmembrane transporter activity (77). Perhaps DNA methylation is associated with ion channels and the immune microenvironment, and *CD3E* is a bridge between the three. There are many studies on the regulation of miRNA transcription by RNA pol II and glioma. Some studies have found that overexpression of EGR-1 may participate in the recruitment of RNA pol II to the GDNF promoter in a non-binding manner, and thus is involved in the regulation of GDNF transcription in glioma cells. This regulation depends on histone hyperacetylation of the GDNF promoter (78). Whether *CD3E* is related to this will be the focus of future investigations. Some studies have found that the ion glutamatergic synapse is associated with medulloblastoma in children (78, 79), while miR-375 also affects the occurrence and development of gastric cancer (80). Therefore, we speculate that *CD3E* and miRNAs may affect the invasion of glioma through the ion glutamate synapse. Some scientists have found that rotenone sensitive NADH ubiquinone oxidoreductase is a key regulatory step in controlling oxidative phosphorylation during the growth period in rat glioma cells (81). Based on the abovementioned bioinformatics analyses of *CD3E*-related core genes in LGG, we found that *CD3E* may be a core gene that can affect the immune microenvironment and tumor purity of LGG in combination with miRNAs, cell respiration, ion channels, and DNA methylation. The role of *CD3E* in brain tumors is completely different from that of extracranial tumors. This may be because *CD3E*, as a core gene, regulates the tumor immune microenvironment in a completely different manner than that of extracranial tumors. However, malignant behavior of CD3E in progression of glioma cell was not elucidated in this study. In follow-up research, we could devote ourselves to exploring the biological malignant function of CD3E and its regulatory mechanism on the tumor immune microenvironment in *in vitro* cell lines, *in vivo* animals, and large-scale multicenter LGG patients.

Overall, we used the ESTIMATE algorithm to determine the TME-related genes in LGG by analyzing LGG samples in TCGA datasets. Through the analysis of LGG samples in GEO, we identified prognostic-related genes in LGG. In our current study, there are still many shortcomings. The first is that the LGG samples we collected are still single-center, the sample size is also small, and they are all Asian patients. We will further expand the samples in the next research. Additionally, we will conduct research on LGG patients in Europe, Africa and other places. The second is that this experiment lacks research on the expression of CD3E in different cell populations in tumor samples. In the next work, we will focus on this direction. The abovementioned studies confirmed that *CD3E* is not only a potential prognostic factor for LGG patients, but also a driving factor for the TME to transform from an immune state to a metabolic state. In the next study, we intend to study the expression of CD3E in different cell populations in LGG to clarify the cell types that express CD3E, as well as how the expression of CD3E in different cell populations affects TME.

## Conclusion

In conclusion, tumor purity has a considerable impact on clinical, genomic and biological status of LGG. *CD3E*, novel membrane immune biomarker deeply affecting tumor purity, may help to evaluate the prognosis and develop individual immunotherapy strategies for LGG patients. Evaluating the ratio of different tumor purity and *CD3E* expression may provide novel insights into the complex structure of the LGG microenvironment and targeted drug development.

## Data Availability Statement

The original contributions presented in the study are included in the article/**Supplementary Material**. Further inquiries can be directed to the corresponding authors.

## Ethics Statement

All of the study designs and test procedures were performed in accordance with the Helsinki Declaration II. The Ethics approval and participation consent of this study was approved and agreed by the ethics committee of Affiliated Hospital of Youjiang Medical College for Nationalities (Baise, Guangxi province, China).

## Author Contributions

HNH, WL, and CL came up with the design and conception. The data analysis and visualization were conducted by WX, HDH, and XL. YW and WL conducted cell line experiments. The original writing of the draft and its editing were by SC, JW, WL, and HZ. All authors contributed to the article and approved the submitted version.

## Funding

This study was supported by grants from: 1. 2020 Guangxi Zhuang Autonomous Region Health Committee self-funded scientific research project, project number 20201558, 2. In 2020, the general project of high-level talent scientific research project of the Affiliated Hospital of Youjiang Medical College for Nationalities (the young and middle-aged backbone talent project), contract number Y202011702, 3. 2021 Guangxi University’s young and middle-aged teachers’ basic research ability improvement project, project number 2021KY0542, and 4. The self-financing project of Guangxi Medicine and Health, project number Z20180200.

## Conflict of Interest

The authors declare that the research was conducted in the absence of any commercial or financial relationships that could be construed as a potential conflict of interest.

## Publisher’s Note

All claims expressed in this article are solely those of the authors and do not necessarily represent those of their affiliated organizations, or those of the publisher, the editors and the reviewers. Any product that may be evaluated in this article, or claim that may be made by its manufacturer, is not guaranteed or endorsed by the publisher.

## Supplementary Material

Supplementary Figure 1 The relationship between *CD3E* expression and survival of UVM patients was shown.

Supplementary Figure 2 The differential expression of cd3e was investigated according to the histological subtypes of LGG. The expression of CD3E in astrocytoma (n = 194) was significantly higher than that in oligoastrocytoma (n = 130, *P* = 6.436000e-04) or oligodendroglioma (n = 130, *P* = 6.418700e-04).

Supplementary Figure 3 A subgroup analysis of different clinical characteristics on clinical data to eliminate clinical bias was performed.

Supplementary Figure 4 Relations between abundance of tumor-infiltrating lymphocytes and expression, copy number, methylation, or mutation of *CD3E*.

Supplementary Figure 5 The relationship between the abundance of tumor infiltrating lymphocytes and the expression, copy number, and methylation or mutation of CD3E in LGG was analyzed.

Supplementary Figure 6 TIDE algorithm to study the effect of CD3E on LGG patients receiving immune checkpoint inhibitor therapy.

Supplementary Figure 7 The expression distribution of CD3E gene in different tumor tissues was shown. The horizontal axis represented different groups of samples, and the vertical axis represented the expression distribution of the gene.

## Abbreviations

AHYMUN, Affiliated Hospital of YouJiang Medical University for Nationalities; GEO, Gene Expression Omnibus; GO, Gene Ontology; GSEA, Gene Set Enrichment Analysis; HR, hazard ratio; IHC, immunohistochemistry; KEGG, Kyoto Encyclopedia of Genes and Genomes; LGG, Low grade glioma; OS, overall survival; PPI, Protein-protein interaction; TCGA, the Cancer Genome Atlas; TIC, tumor-infiltrating immune cells; TME, tumor microenvironment.
