# Supplementary figures and images for: Malignant Tumor Purity Reveals the Driven and Prognostic Role of CD3E in Low-Grade Glioma Microenvironment
Source: Front Oncol. 2021 Sep 7;11:676124. doi: 10.3389/fonc.2021.676124 (PMC8454269; doi:10.3389/fonc.2021.676124)

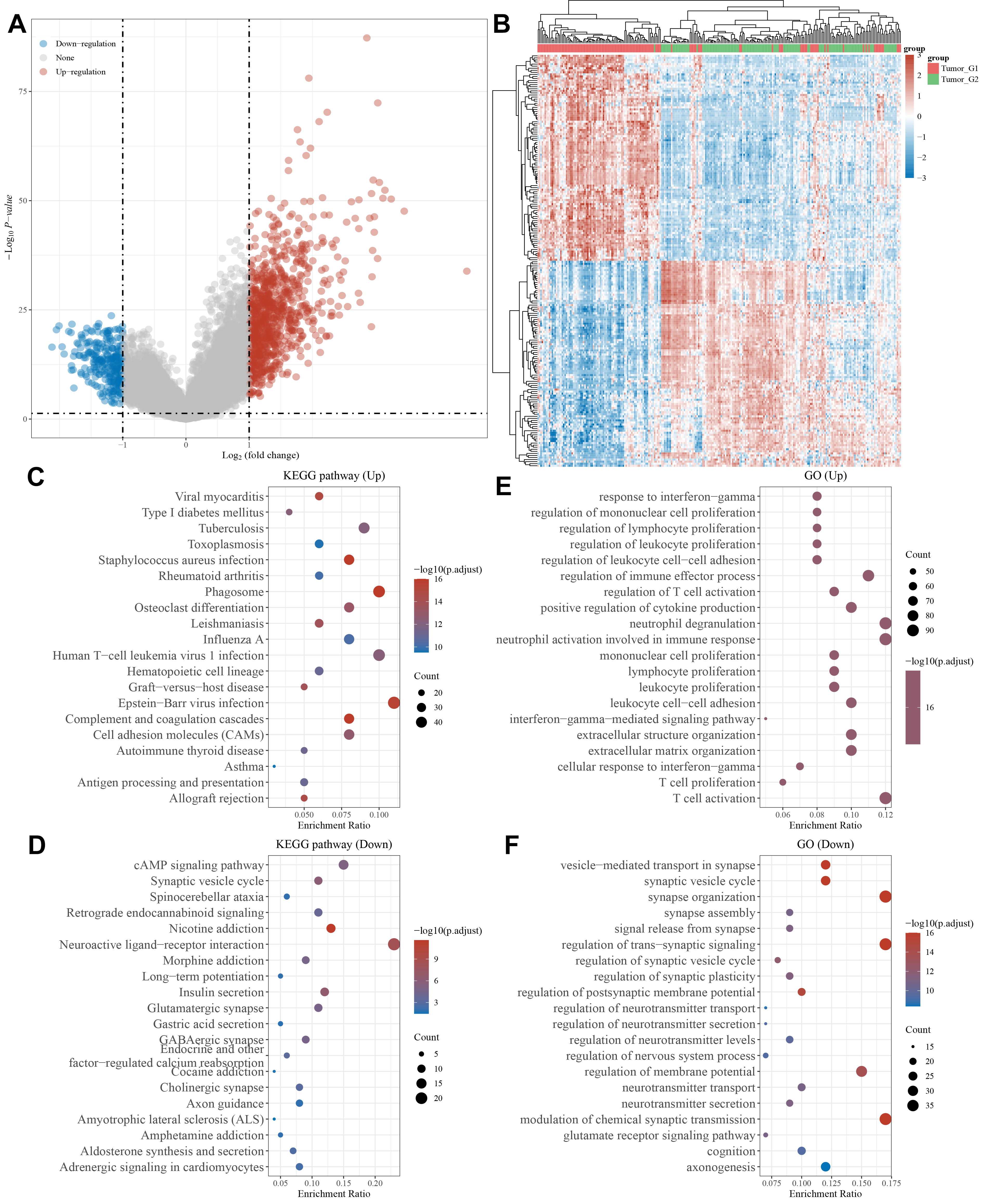

Supplement: Supplementary Figure 1 — The relationship between CD3E expression and survival of UVM patients was shown. [file Image_1.tif]

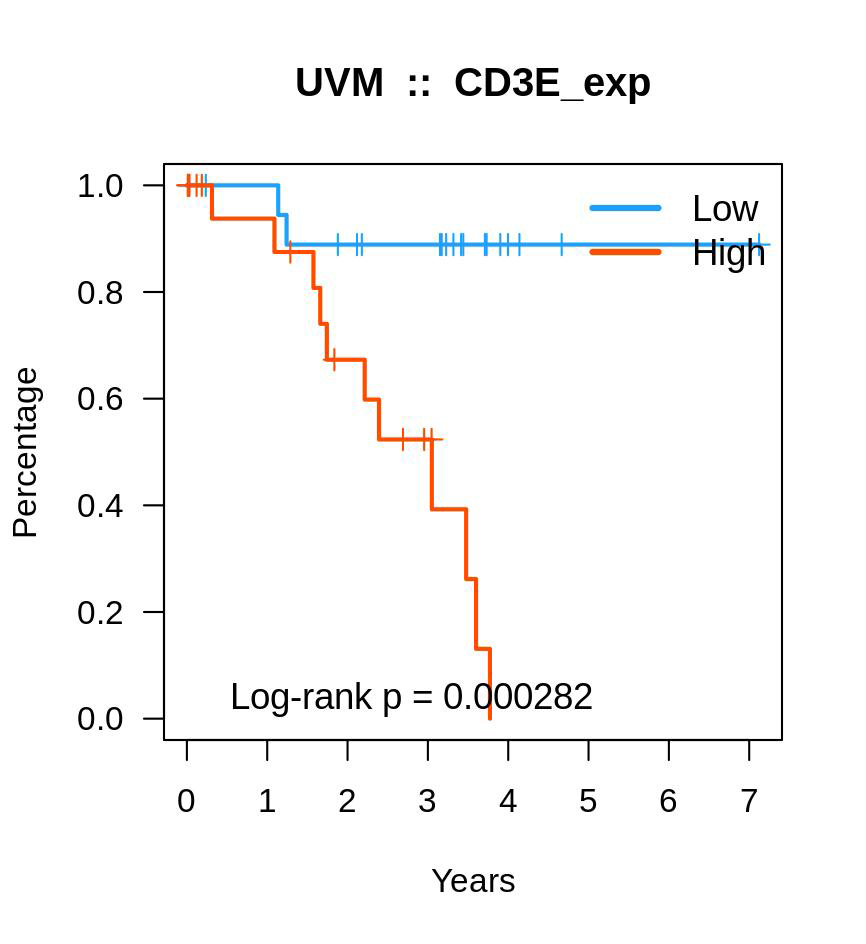

Supplement: Supplementary Figure 2 — The differential expression of cd3e was investigated according to the histological subtypes of LGG. The expression of CD3E in astrocytoma (n = 194) was significantly higher than that in oligoastrocytoma (n = 130, P = 6.436000e-04) or oligodendroglioma (n = 130, P = 6.418700e-04). [file Image_2.tif]

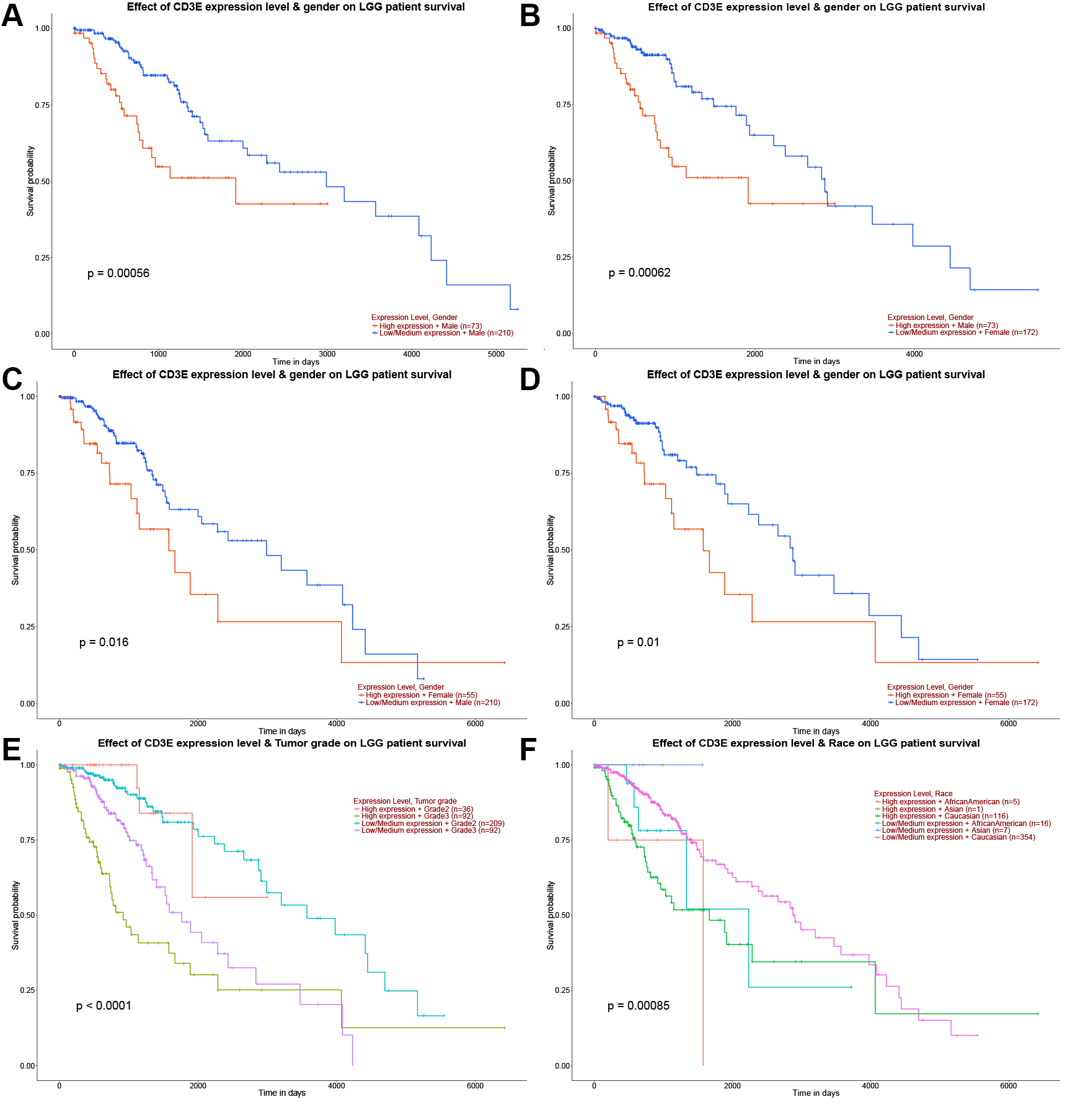

Supplement: Supplementary Figure 3 — A subgroup analysis of different clinical characteristics on clinical data to eliminate clinical bias was performed. [file Image_3.tif]

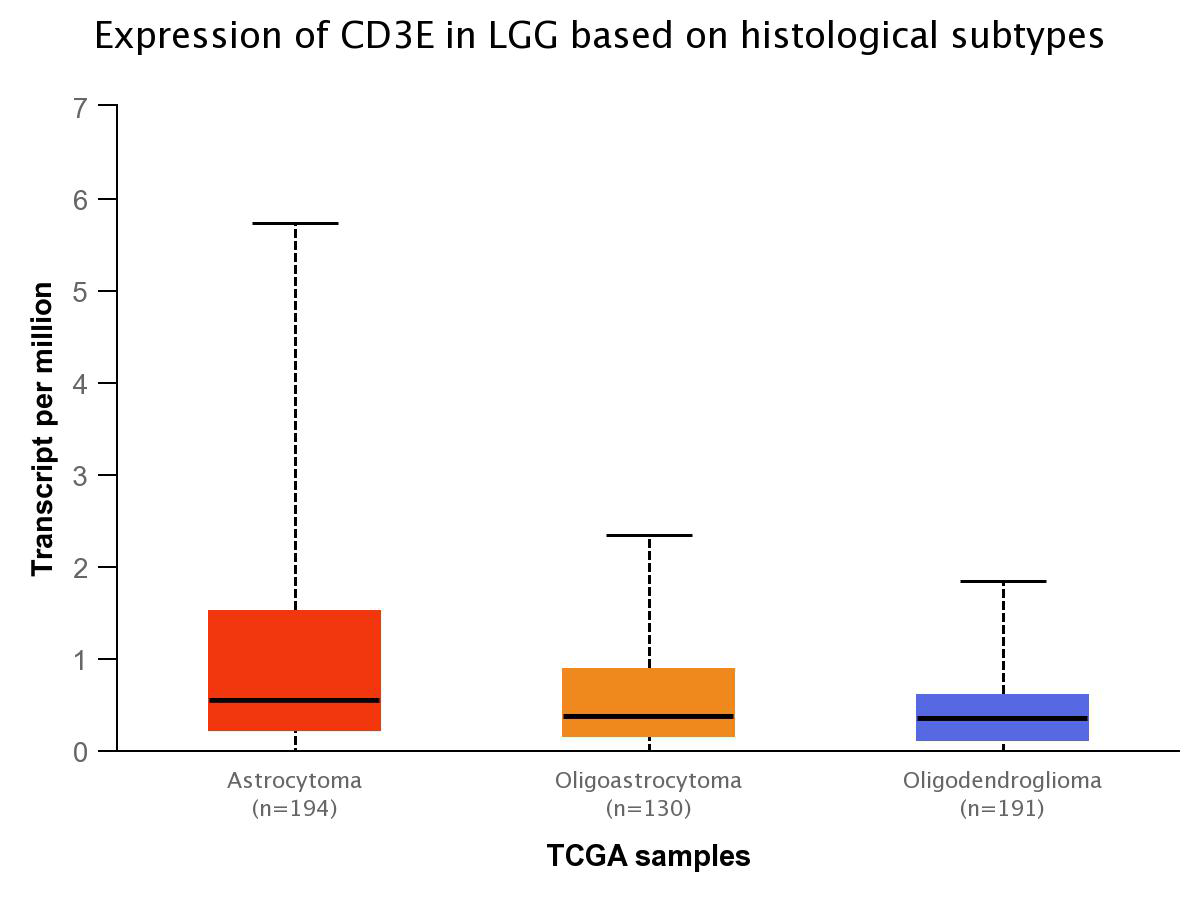

Supplement: Supplementary Figure 4 — Relations between abundance of tumor-infiltrating lymphocytes and expression, copy number, methylation, or mutation of CD3E. [file Image_4.tif]

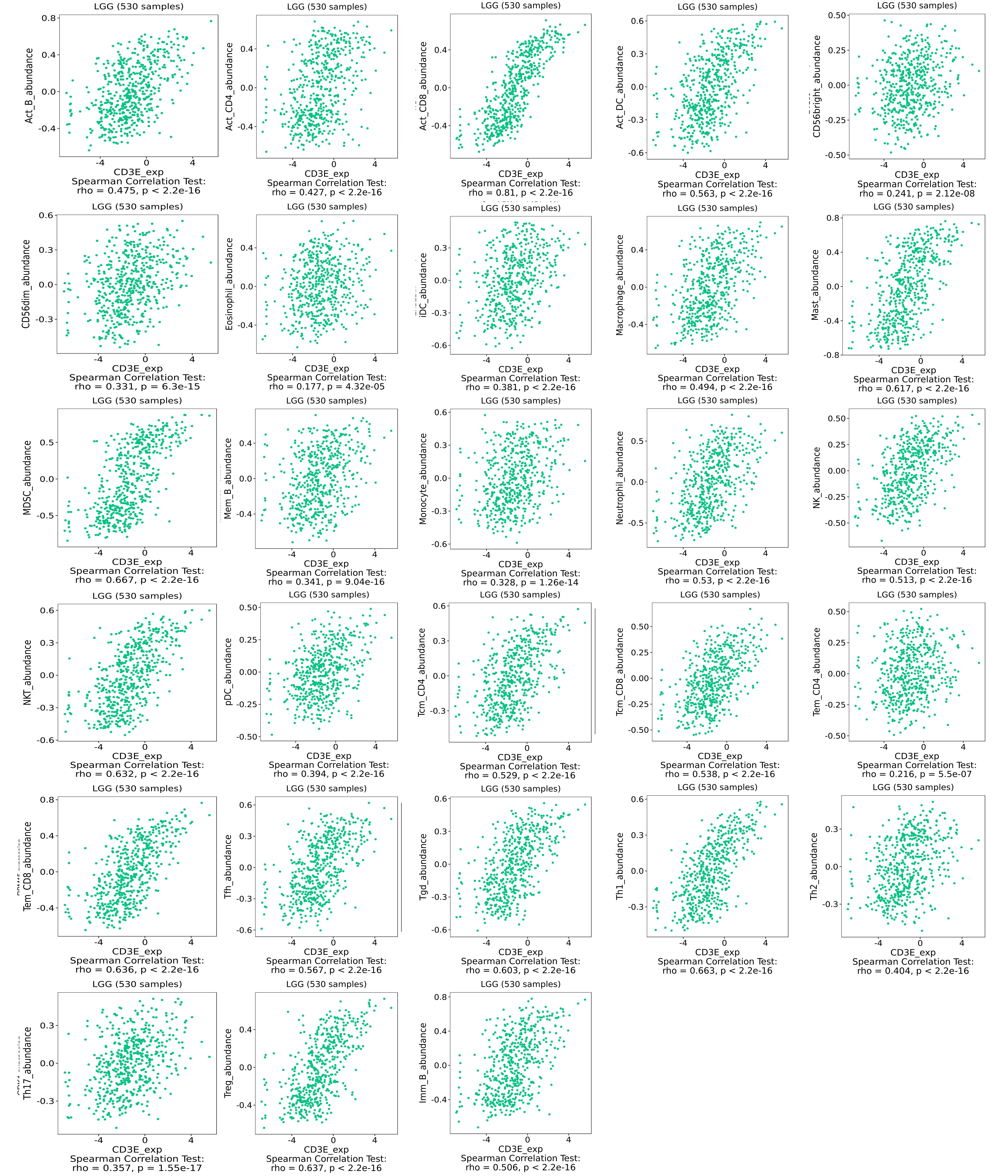

Supplement: Supplementary Figure 5 — The relationship between the abundance of tumor infiltrating lymphocytes and the expression, copy number, and methylation or mutation of CD3E in LGG was analyzed. [file Image_5.tif]

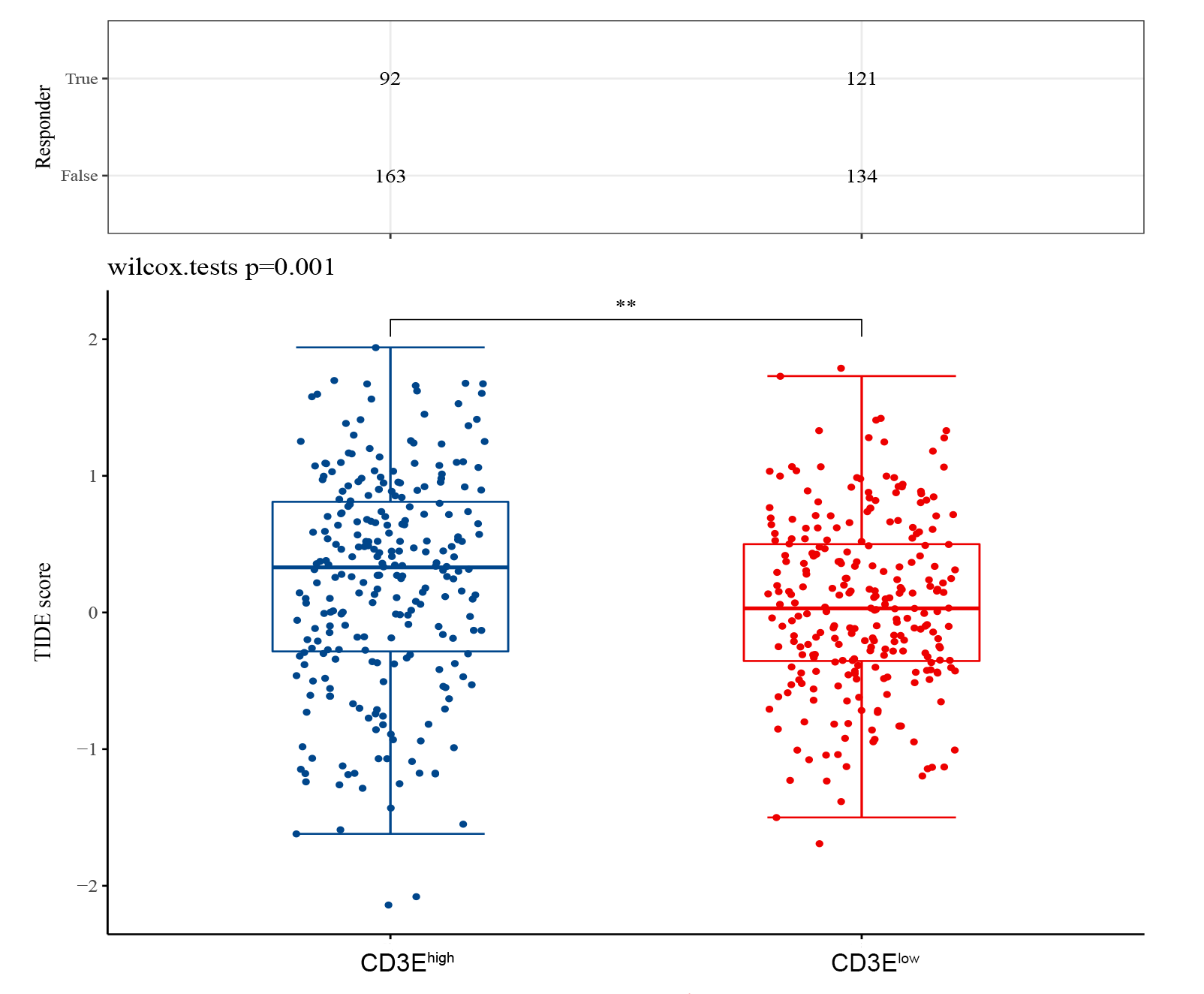

Supplement: Supplementary Figure 6 — TIDE algorithm to study the effect of CD3E on LGG patients receiving immune checkpoint inhibitor therapy. [file Image_6.tif]

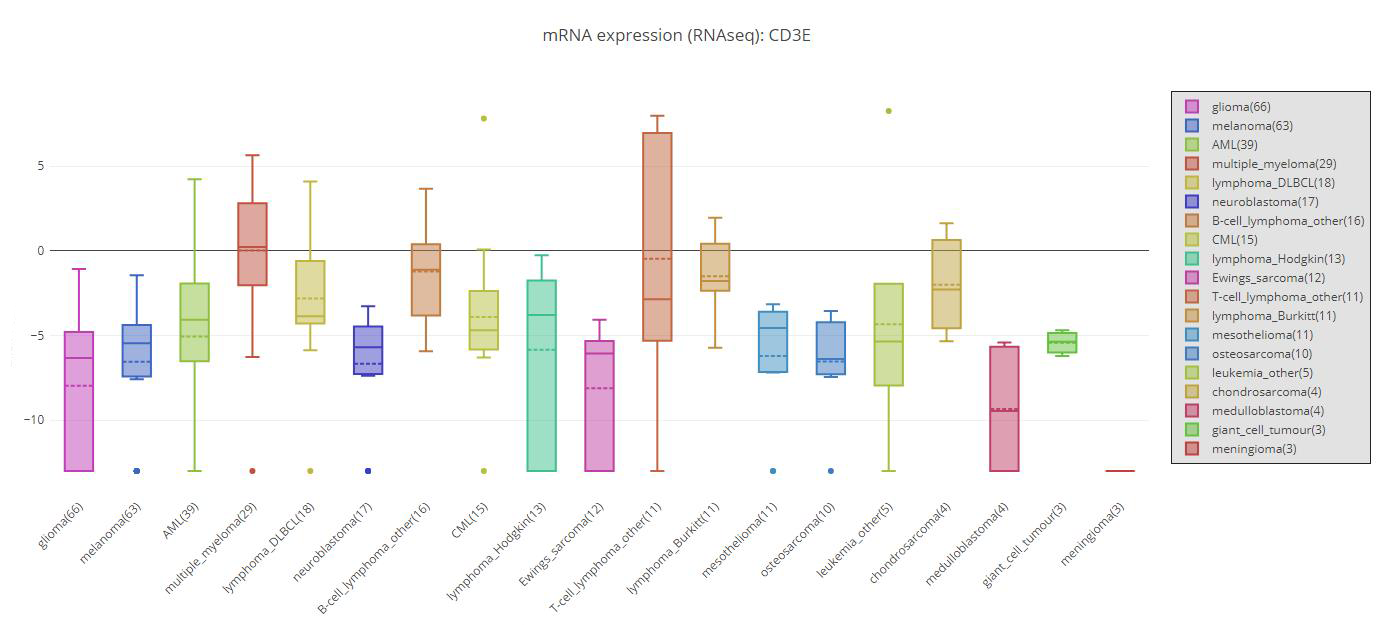

Supplement: Supplementary Figure 7 — The expression distribution of CD3E gene in different tumor tissues was shown. The horizontal axis represented different groups of samples, and the vertical axis represented the expression distribution of the gene. [file Image_7.tif]
